# Supplementary material for: The highly dynamic nature of bacterial heteroresistance impairs its clinical detection
Source: Commun Biol. 2021 May 5;4:521. doi: 10.1038/s42003-021-02052-x (PMC8099907; doi:10.1038/s42003-021-02052-x)
Supplement: Supplementary file 1 — Supplementary Information [file 42003_2021_2052_MOESM1_ESM.pdf]

SUPPLEMENTARY INFORMATION

The highly dynamic nature of bacterial heteroresistance impairs its clinical detection

Cátia Pereira[1], Jimmy Larsson[2], Karin Hjort[1], Johan Elf[2] and Dan I. Andersson[1*]

[1]Department of Medical Biochemistry and Microbiology, Uppsala University, Sweden

[2]Department of Cell and Molecular Biology, Uppsala University, Sweden

Supplementary Table 1. MIC increase and Clinical Breakpoints for the populations evolved in the presence of sub-MIC antibiotic concentrations and respective MIC of the wild-type parental strain. Determinations of MICs were based on 3 biological and 1 technical replicates.

Antibiotic	Clinical Breakpoint (mg/L) [2]	MIC of the wild-type susceptible strain (mg/L)	Clinical Breakpoint ($xMIC_{WT}$)[2]	MIC Increase ($xMIC_{WT}$) in the population evolved at $1xMIC_{WT}$
Tobramycin	4	3.0	1.3	3 – 8
Tetracycline	16	12.0	2.0	4
Cefepime	4	0.19	21	340 – 2723
Piperacillin-Tazobactam	16	8.0	1.3	5.3

Supplementary Table 2. Statistical analysis of relative exponential growth rates differences between isolates with different copy number. Mean value of relative exponential growth rate per copy number and respective *p*-value obtained from the *T*-test performed to calculate the significance of relative exponential growth rate differences between copy numbers.

Antibiotic Concentration ($xMIC_{WT}$)	*E. coli* DA33135			*S.* Typhimurium DA34827		
	Mean Relative Exponential Growth Rate		*p*-value	Mean Relative Exponential Growth Rate		*p*-value
	CN = 2.3	CN = 5.1		CN = 3.5	CN = 9.4	
1/64	0.8903333	0.8755000	0.03138	0.9953166	0.9664259	0.00747
1/16	0.9198667	0.8879333	0.00349	0.9982810	0.9817932	0.07571
1/8	0.9403667	0.9017667	0.09967	1.0134308	0.9916848	0.06182
1/4	0.9428000	0.9007667	0.00497	1.017661	1.009225	0.2241
1/2	0.9030	0.8778	0.06459	0.9811492	0.9863017	0.641
1	0.7585667	0.8705667	0.00536	0.9036811	0.9563884	0.01884
2	0.7305667	0.8078667	0.07548	0.6775856	0.9033340	0.01413
4	0.6250667	0.6167667	0.6729	-	-	-

Supplementary Table 3. Recombination rate, cost of duplication and calculated cost (%) per kbp of DNA estimated by the model simulation and fitness cost (%) per kbp of DNA.

	Strain	Size of the amplified unit (kbp)	Estimated parameters			Fitness cost (%) /kbp of DNA (Batch Culture)
			Recombination rate (k_{rec})	Cost of duplication (s)	Calculated cost (%) /kbp of DNA	
Tobramycin	DA69068	27.7	0.0029	-0.012	0.043	0.0237
	DA69069	27.7	0.0030	-0.017	0.061	0.0395
Tetracycline	DA69071	9.7	0.0032	-0.003	0.031	0.1135
	DA69073	9.7	0.0032	-0.004	0.041	0.0463
Cefepime	DA69074	10.4	0.0029	-0.001	0.010	0.0232
	DA69075	10.4	0.0037	-0.004	0.038	0.0414
	DA69076	10.4	0.0034	-0.004	0.038	0.0583
Piperacillin-Tazobactam	DA69077	3.5	0.0025	-0.002	0.057	0.1154
	DA69078	3.5	0.0029	-0.005	0.143	0.0539
	DA69079	3.5	0.0020	-0.001	0.029	0.0501

Supplementary Table 4. List of strains used in this study.

DA number	Species	Genotype	Reference/source	Comment
DA33135	*E. coli*	Chromosome: 4.96 Mbp, plasmids 139kbp pDA33135-139, 70kbp pDA33135-70	[1]	Clinical isolate ECO-005
DA34827	*S. enterica* Var. Typhimurium	Chromosome: 4.93 Mbp	The public health agency of Sweden, received from Cecilia Järnberg (unpublished)	Clinical Isolate from feces, Sweden
DA34833	*S. enterica* Var. Typhimurium	Chromosome: 4.93 Mbp, plasmid 94 kbp pDA34833-94	The public health agency of Sweden, received from Cecilia Järnberg (unpublished)	Clinical Isolate from feces, Sweden
DA61218	*E. coli*	Chromosome: 5.1 Mbp, plasmid 116 kbp pDA61218-116	Received from Uppsala University Hospital (unpublished)	Clinical isolate, Sweden
DA63680	*E. coli*	-	Received from Eva Tano (unpublished)	Clinical isolate collected from patients with sepsis during 2014-2015, Sweden
DA63702	*E. coli*	-	Received from Eva Tano (unpublished)	Clinical isolate collected from patients with sepsis during 2014-2015, Sweden
DA63818	*E. coli*	-	Received from Eva Tano (unpublished)	Clinical isolate collected from patients with sepsis during 2014-2015, Sweden
DA63834	*E. coli*	-	Received from Eva Tano (unpublished)	Clinical isolate collected from patients with sepsis during 2014-2015, Sweden
DA63836	*E. coli*	-	Received from Eva Tano (unpublished)	Clinical isolate collected from patients with sepsis during 2014-2015, Sweden
DA63870	*E. coli*	-	Received from Eva Tano (unpublished)	Clinical isolate collected from patients with sepsis during 2014-2015, Sweden

DA63874	*E. coli*	-	Received from Eva Tano (unpublished)	Clinical isolate collected from patients with sepsis during 2014-2015, Sweden
DA63882	*E. coli*	-	Received from Eva Tano (unpublished)	Clinical isolate collected from patients with sepsis during 2014-2015, Sweden
DA63918	*E. coli*	-	Received from Eva Tano (unpublished)	Clinical isolate collected from patients with sepsis during 2014-2015, Sweden
DA69068	*E. coli*	Chromosome: 4.96 Mbp, plasmids 139kbp pDA69068-139: ampl 27.7 kbp, 70kbp pDA33135-70	This study	DA33135 selected at 3.0mg/L tobramycin
DA69069	*E. coli*	Chromosome: 4.96 Mbp, plasmids 139kbp pDA69069-139: ampl 27.7 kbp, 70kbp pDA33135-70	This study	DA33135 selected at 3.0mg/L tobramycin
DA69070	*E. coli*	Chromosome: 4.96 Mbp, plasmids 139kbp pDA69070-139: ampl 27.7 kbp, 70kbp pDA33135-70	This study	DA33135 selected at 3.0mg/L tobramycin
DA69071	*S. enterica* Var. Typhimurium	Chromosome: 4.93 Mbp ampl 9.7 kbp	This study	DA34827 selected at 12.0mg/L tetracycline
DA69072	*S. enterica* Var. Typhimurium	Chromosome: 4.93 Mbp ampl 9.7 kbp	This study	DA34827 selected at 12.0mg/L tetracycline
DA69073	*S. enterica* Var. Typhimurium	Chromosome: 4.93 Mbp ampl 9.7 kbp	This study	DA34827 selected at 12.0mg/L tetracycline
DA69074	*S. enterica* Var. Typhimurium	Chromosome: 4.93 Mbp ampl 10.4 kbp, plasmid 94 kbp pDA69074-94	This study	DA34833 selected at 0.094mg/L cefepime
DA69075	*S. enterica* Var. Typhimurium	Chromosome: 4.93 Mbp ampl 10.4 kbp, plasmid 94 kbp pDA69074-94	This study	DA34833 selected at 0.094mg/L cefepime
DA69076	*S. enterica* Var. Typhimurium	Chromosome: 4.93 Mbp ampl 10.4 kbp, plasmid 94 kbp pDA69074-94	This study	DA34833 selected at 0.094mg/L cefepime
DA69077	*E. coli*	Chromosome: 5.1 Mbp, plasmid 116 kbp: pDA69077-116 ampl 3.5 kbp	This study	DA61218 selected at 64mg/L piperacillin-tazobactam
DA69078	*E. coli*	Chromosome: 5.1 Mbp, plasmid 116 kbp: pDA69077-116 ampl 3.5 kbp	This study	DA61218 selected at 64mg/L piperacillin-tazobactam
DA69079	*E. coli*	Chromosome: 5.1 Mbp, plasmid 116 kbp: pDA69077-116 ampl 3.5 kbp	This study	DA61218 selected at 64mg/L piperacillin-tazobactam

Supplementary Table 5. List of primers used for the digital droplet PCR.

Primer	Sequence (5'-3')	Description
aac(3) – F	AATCCGATGCCGTTTTCCAG	Oligo binding to the putative resistance gene *aac(3)-IId* in the plasmid pDA33135-139
aac(3) – R	AAACTCCGTTACCGCATTGC	Oligo binding to the putative resistance gene *aac(3)-IId* in the plasmid pDA33135-139
plasmid6 – F	CTCCCTGAGAAGAATGGCCA	Oligo binding to the plasmid pDA33135-139 (used to determine plasmid copy number)
plasmid6 – R	GCATATGGTGACGCTGATCC	Oligo binding to the plasmid pDA33135-139 (used to determine plasmid copy number)
lpp – F	CTACTCTGCTGGCAGGTTGC	Oligo binding to the chromosome DA33135
lpp – R	CACGAGCTGCGTCATCTTTAG	Oligo binding to the chromosome DA33135
tet(A)2 – F	GCGGTCGGTATTGTCTTCAC	Oligo binding to the putative resistance gene *tet(A)* in the chromosome DA34827
tet(A)2 – R	GGATGCAGAAGTAGAACGCG	Oligo binding to the putative resistance gene *tet(A)* in the chromosome DA34827
glpK – F	TGCATCAGGAAGTTGTTGGC	Oligo binding to the chromosome DA34827
glpK – R	AATCACATTATCCGCGCGAC	Oligo binding to the chromosome DA34827
blaCARB – F	AAGTAGGGCAGGCAATCACA	Oligo binding to the putative resistance gene *blaCARB* in the chromosome DA34833
blaCARB – R	GACGAGTCTCTTTGTCCCCA	Oligo binding to the putative resistance gene *blaCARB* in the chromosome DA34833
kdpB – F	ATCACAATGCTGCGACCTTC	Oligo binding to the chromosome DA34833
kdpB – R	GCCAGGCGTCCGATTTTATT	Oligo binding to the chromosome DA34833
blaSHV(1) – F	CTATCCGGGCAATCGTTGAG	Oligo binding to the putative resistance gene *blaSHV* in the plasmid pDA61218-116
blaSHV(1) – R	TTTTCATTCCACGGGTCAGG	Oligo binding to the putative resistance gene *blaSHV* in the plasmid pDA61218-116
plasmid3 – F	TTGCCGACTACCTTGGTGAT	Oligo binding to the plasmid pDA61218-116 (used to determine plasmid copy number)
plasmid3 – R	CCCAGTATCAGCCCGTCATA	Oligo binding to the plasmid pDA61218-116 (used to determine plasmid copy number)
rplT – F	GCGATATCAGCCAGGATCTTAC	Oligo binding to the chromosome DA61218
rplT – R	GTCGTCAACGTAAGCGTCAG	Oligo binding to the chromosome DA61218

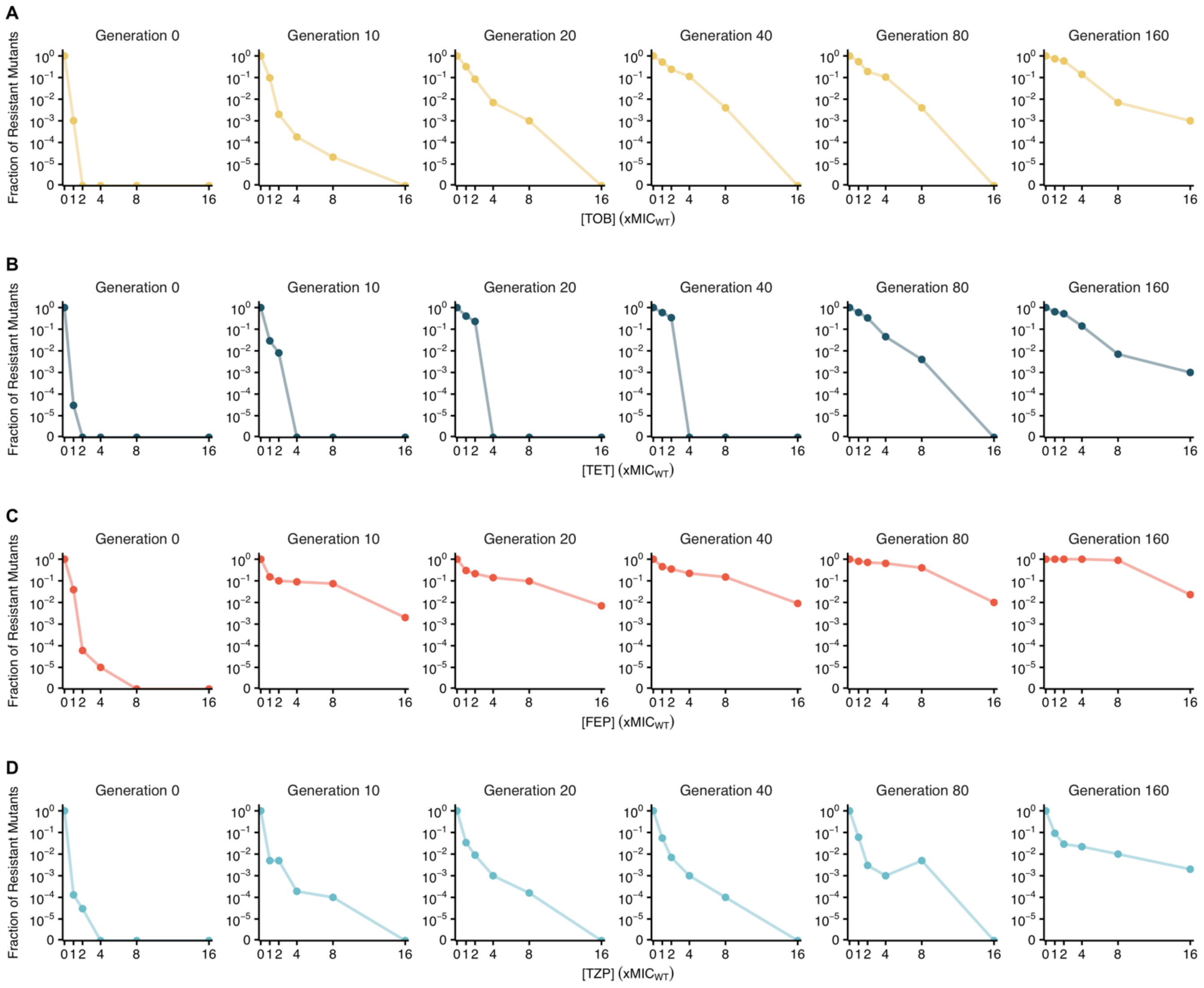

Supplementary Figure 1. Population Analysis Profile of resistant sub-populations selected at 1xMIC$_{WT}$. Populations were evolved from (A) *E. coli* DA33135 in the presence of tobramycin, (B) *S.* Typhimurium DA34827 in the presence of tetracycline (C) *S.* Typhimurium DA34833 in the presence of cefepime and (D) *E. coli* DA61218 in the presence of piperacillin-tazobactam. One serially passaged lineage per antibiotic was plated on Mueller-Hinton agar containing increasing concentrations of antibiotic and the fraction of resistant mutants was determined.

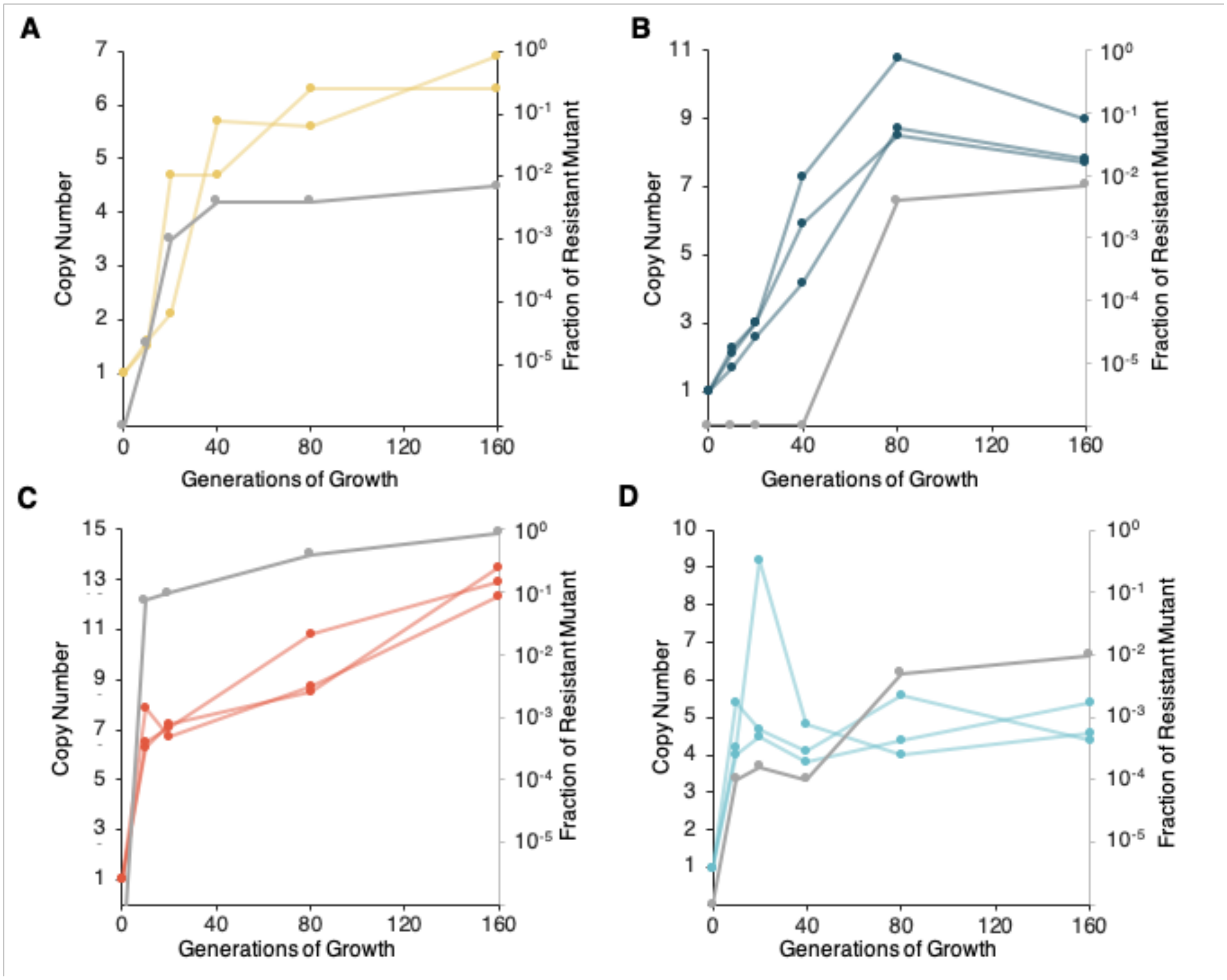

Supplementary Figure 2. Copy number and selection at 1xMIC$_{WT}$. Subpopulations were evolved from (A) *E. coli* DA33135 resistant to 8xMIC$_{WT}$ of tobramycin, (B) *S.* Typhimurium DA34827 resistant to 8xMIC$_{WT}$ tetracycline (C) *S.* Typhimurium *DA34833* resistant to 8xMIC$_{WT}$ cefepime and (D) *E. coli* DA61218 resistant to 8xMIC$_{WT}$ piperacillin-tazobactam. In grey, the fraction of resistant mutants resistant to the antibiotic tested for selection. In color, the gene copy number of the mutants selected at 1xMIC$_{WT}$ of the same antibiotics. Two independent clones were analysed for the evolution of *E. coli* DA33135 in the presence of tobramycin at 1xMIC$_{WT}$ of the susceptible strain; three independent clones were analysed for all other experiments.

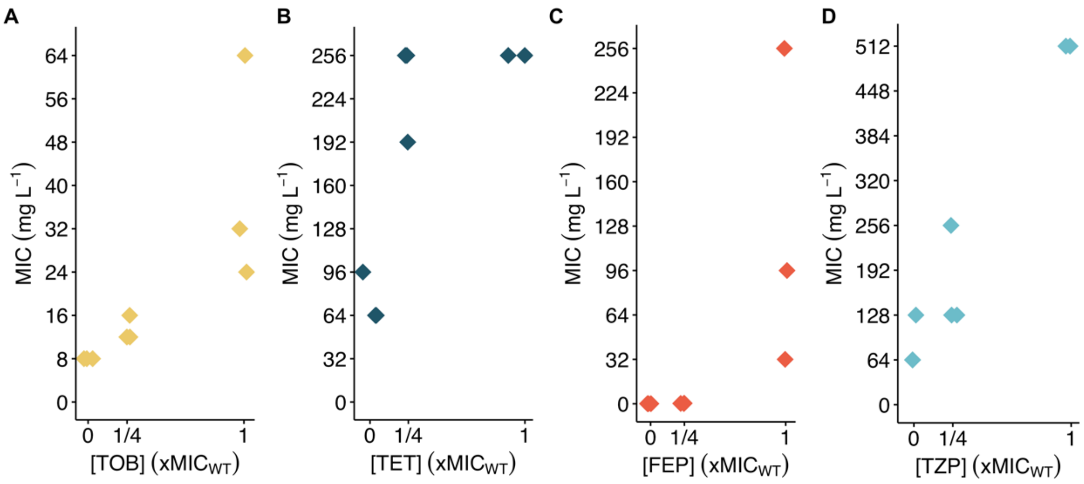

Supplementary Figure 3. MIC of the populations as a function of selective antibiotic concentrations. Tobramycin (A), tetracycline (B), cefepime (C) and piperacillin-tazobactam. The MIC was measured for three biological replicates of the wild-type strains (0x the MIC of the susceptible strain) and the populations were evolved at 1/4x and 1x the MIC of the susceptible strain for 80 generations. Determinations of MICs were based on 3 biological and 1 technical replicates.

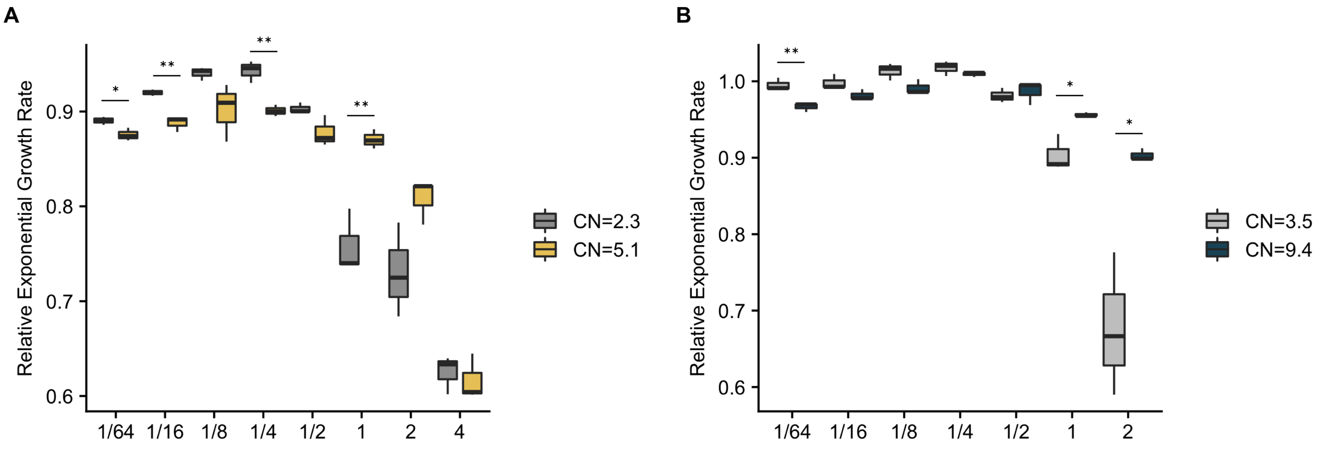

Supplementary Figure 4. Mean relative exponential growth rate of isolates with different copy numbers at increasing antibiotic concentrations. (A) tobramycin and (B) tetracycline. Growth measured for independent clones evolved from the heteroresistant parental strains (A) *E. coli* DA33135 and (B) *S.* Typhimurium DA34827. The growth rates are relative to the growth rate of the heteroresistant parental isolates in the absence of selection. *T*- tests were performed to calculate the significance of the differences in relative exponential growth rate observed between isolates with different copy number, where * indicates a *p*-value < 0.05 and **indicates a *p*-value < 0.01.

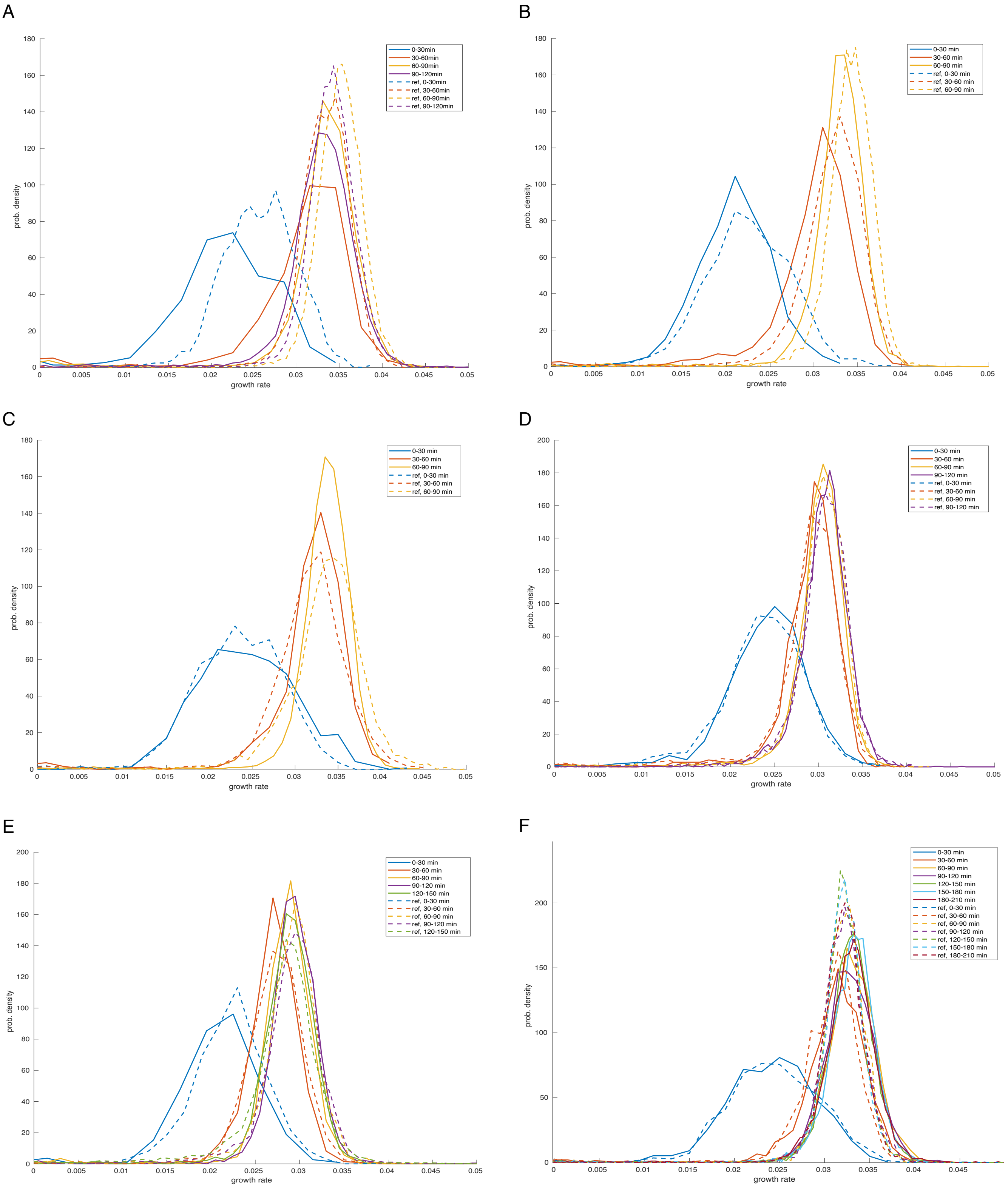

Supplementary Figure 5. Single-cell growth rate measurements. Solid lines show the distribution of growth rate for the sample cells measured at different 30-minute time intervals (each color represents a time interval) and dashed lines show the distribution of growth rate for the reference single-cell growth rates. Growth rates measured for samples DA69068 (A), DA69069 (B), DA69070 (C), DA69072 (D), DA69074 (E) and DA69078 (F).

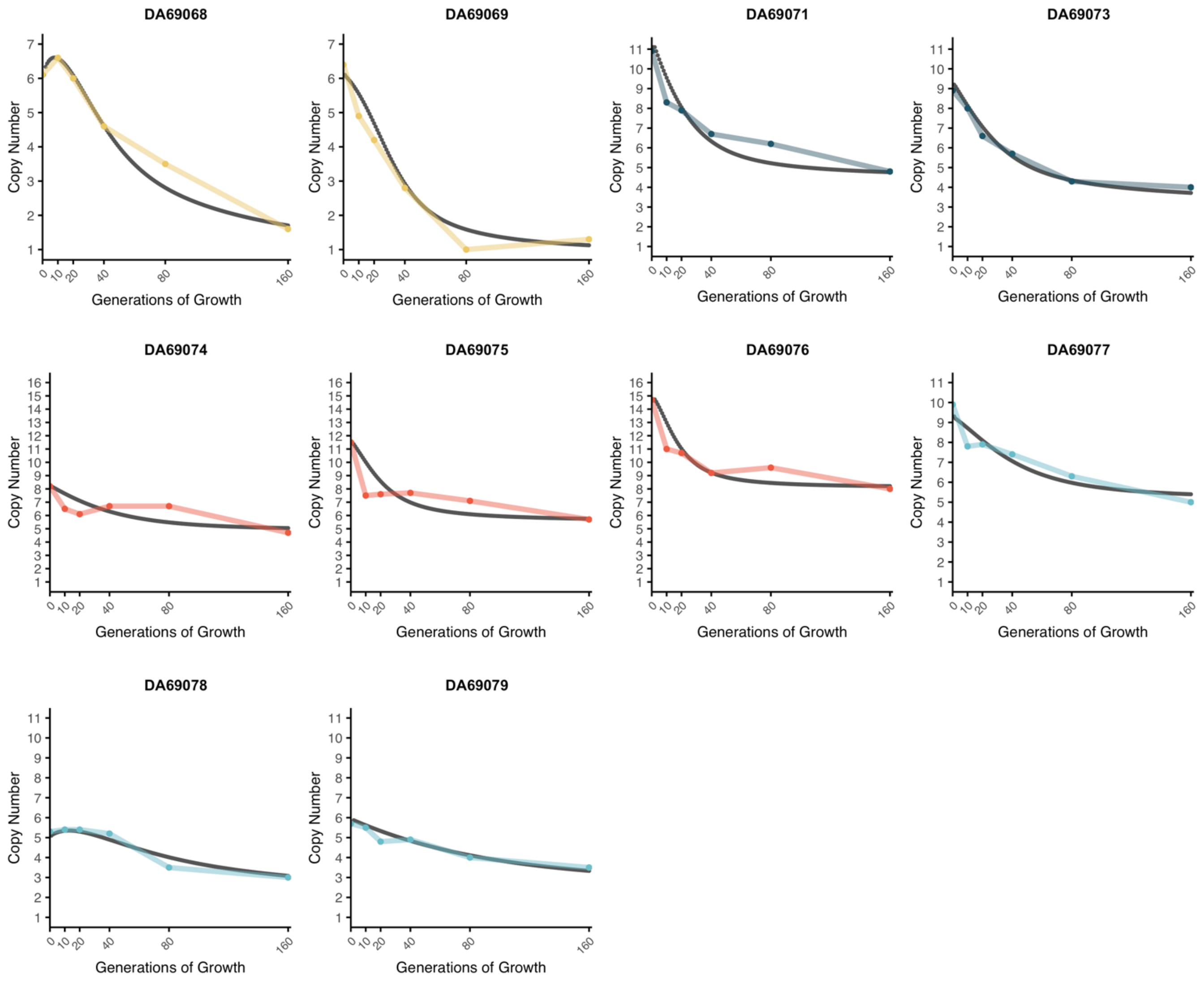

Supplementary Figure 6. Determination of fitness cost and recombination rates of amplified units for loss of gene amplifications using the model for homologous recombination. Colored lines show the copy number as a function of generations of growth for the experimental rate of loss of each mutant in which no additional mutations were found. Yellow, blue, orange and turquoise represent isolates evolved from the parental strain *E. coli* DA33135 evolved in the presence of tobramycin, *S*. Typhimurium DA34827 in the presence of tetracycline, *S.* Typhimurium DA34833 in the presence of cefepime and *E. coli* DA61218 in the presence of piperacillin-tazobactam, respectively. Gray lines represent the simulated results using the k_{rec} and *s* parameters that best fitted the model.

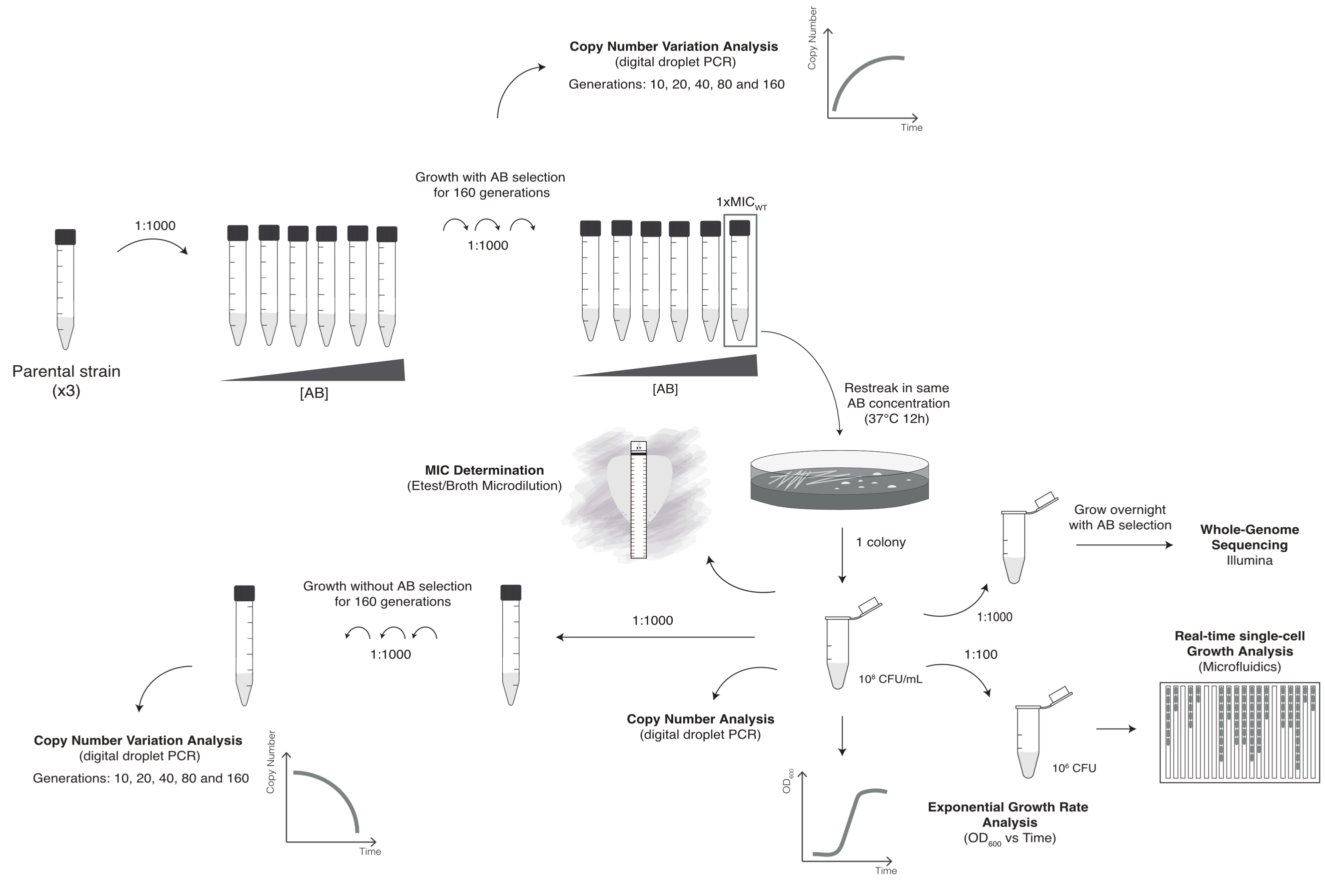

Supplementary Figure 7. Schematic outline of the method used for the selection of resistant subpopulations in the presence of sub-lethal antibiotic concentrations, characterization of isolates with variable initial copy number and evolution in the absence of selection.

References

1. Brolund, A., Franzén, O., Melefors, O., Tegmark-Wisell, K. & Sandegren, L. Plasmidome-analysis of ESBL-producing escherichia coli using conventional typing and high-throughput sequencing. *PLoS One* **8**, e65793–e65793 (2013).
2. *The European Committee on Antimicrobial Susceptibility Testing. Breakpoint tables for interpretation of MICs and zone diameters, version 10.0, 2020*. http://www.eucast.org/clinical_breakpoints/.
